# Comparison of Periodontal Status in Gingival Oral Lichen Planus Patients and Healthy Subjects

**DOI:** 10.1155/2012/561232

**Published:** 2012-04-18

**Authors:** Arash Azizi, Massoud Rezaee

**Affiliations:** Oral Medicine Department, Islamic Azad University, Dental Branch, Tehran 1668637161, Iran

## Abstract

*Background and Objective*. Oral lichen planus (OLP) is a common chronic mucocutaneous disease. OLP can occur in different oral sites such as gingiva. The purpose of study was to evaluate the periodontal status of OLP patients with desquamative gingivitis (DG) and compare it with that of healthy control. *Methods*. This study was case-control. 32 patients with gingival OLP as a case group and 32 healthy subjects as a control group were selected. The periodontal status of all subjects including plaque index (PI), bleeding on probing (BOP), and clinical attachment level (CAL) was evaluated in both groups. Finally data were analyzed by *t*-test. *Results*. The mean values of periodontal parameters were observed to be higher in case group compared with control group, and this was significant (*P* < 0.05). *Conclusion*. Our results showed that periodontal status is worse in gingival OLP if compared with healthy controls.

## 1. Introduction

Oral lichen (OLP) is a common chronic mucocutaneous disease [1]. The prevalence of OLP varies from 0.1% to about 4% depending on the population sampled [2–4]. OLP is mostly found in middle-aged and elderly patients, the female- to male- ratio is nearly 3 : 2 [5]. OLP is characterized histopathologically by variable epithelial thickness, basal cell destruction, and a band-like infiltrate of mononuclear cells in the lamina propria. In the oral cavity gingival tissues are one of the most common sites for OLP [6].

Gingival OLP is characterized by erythematous lesions, erosions, and ulcers, mainly located on the attached gingival. The presence of epithelial desquamation, erythema, and erosive lesions on the gingival tissue is described as desquamative gingivitis [7–9]. It has been suggested that DG could play a role in increasing the long risk for periodontal tissue breakdown at specific sites [10, 11]. Arduino et al. demonstrated that periodontal status is worse in mucous membrane pemphigoid (MMP) patients with DG [10]. Schellinck et al. explained that patients with MMP have higher index compared to control groups [11]. Results of Lo Russo et al. study indicated that sites where DG lesions are present are not significantly different from sites where DG lesion are absent [8]. This is not the first study about the periodontal involvement and lichen planus, but it is one of the the first investigation about this title.

The purpose of this study was to examine the periodontal status of subjects with gingival oral lichen planus compared to control.

## 2. Methods

 This study was a case-control. Sixty-four patients referred to the Department of Oral Medicine at the Azad University in Iran between 2006 and 2009 were selected. Thirty-two patients with diagnosis gingival oral lichen planus as case group and thirty-two subjects with no oral lichen planus and no history of desquamative gingivitis related to OLP as control group were enrolled in this study. Diagnosis of gingival OLP was made by clinical evaluation and confirmed by histological examination.

Exclusion criteria included (i) history of previous and/or current treatment for desquamative gingivitis and periodontal therapy (surgical and nonsurgical), (ii) less than 20 teeth, and (iii) systemic disease and pregnancy. The case and control subjects were between 30 to 65 years old and did not take any medication or supplement.

An informed consent was taken from each participant (case and control). This study was approved by ethics committee at the Ahwaz Dental School, University of Jundishapur. The oral clinical examination was performed by a single calibrated investigator. Periodontal examination including the following criteria was done. Plaque index (PI) and gingival index (GI), probing depth (PD), bleeding on probing (BOP), and clinical attachment level (CAL) were assessed in both groups.

All periodontal measurements were performed at six sites per tooth. All measurements were performed with a periodontal probe (PCPUNC 15: HU-Fridy Chicago, IL, USA) and the reading was recorded to the nearest 1 mm. PI was used for evaluating the state of dental plaque adhesion and GI was used for evaluating the spread and severity of gingival margin inflammation. CAL measured the distance from the periodontal pocket depth to the cementoenamel junction.

PI and GI were evaluated based on Silness and Loe's method [12]. PD was measured from the gingival margin to the base of the probable pocket. BOP was evaluated based on the presence or absence of gingival bleeding on probing [12]. We do not have any pictures about this patients, because we did not evaluate any treatment about desquamative gingivitis. The mean values of the individual subjects were analyzed by *t*-test. *P* values ≤0.05 were considered to be statistically significant. For analyzing of data, we used Spss 17 statistical software.

## 3. Results

 The mean and standard deviation age of the case was 45.1 ± 7.2 and that of the control group was 48.4 ± 4.5. There was no significant difference in both groups. The mean values of periodontal parameters are shown in Table 1.

All PI, GI, PD, BOP, and CAL parameters were significantly higher in the case group compared with the control group, and the difference was significant (*P* < 0.05).

## 4. Discussion

The purpose of the study was to compare periodontal parameters in case and control groups. Our results showed that periodontal parameters were higher in case group compared to the control group. This increase may be due to several factors. It seems reasonable to believe that patients with desquamative gingivitis resulting from OLP may have impaired capacity to perform efficient oral hygiene practices hence the increased gingival inflammation levels and periodontal break down. In addition, discomfort caused by gingival lesions could predispose patients to visit less their dentists on a regular basis. OLP gingival lesions are usually persistent and painful, thus limiting efficient teeth brushing, this leads to plaque accumulation and could increase the possibility of long-term periodontal diseases [11].

Our results are in agreement with those of Arduino et al. [10], and Newman et al. [13]. Newman et al. found that periodontal status in pemphigus vulgaris patients might contribute to development and progression of periodontitis [13].

 Arduino et al. showed that periodontal status is worse in mucous membrane pemphigoid (MMP) patients with desquamative gingivitis if compared with healthy controls due to a substantial difference in oral hygiene [10]. They demonstrated that oral health should be promoted in mucous membrane pemphigoid [10].

There is no enough study about correlation of gingival oral lichen planus and periodontal parameters, but there is some study about periodontal parameters in mucous membrane pemphigoid and pemphigus vulgaris with desquamative gingivitis such as Arduino et al. [10], Schellinck et al. [11], and Akman et al. [12] studies, with those of Lo Russo et al. [8] that found no correlation between desquamative gingivitis (DG) and periodontal status [8]. This difference may be due to the fact that in their studies number of patients with desquamative gingivitis was little. They evaluated only 12 patients with DG, but we evaluate 64 patients with DG oral lichen planus and healthy control subjects. Schellinck et al. and Tricomo et al. demonstrated that patients with MMP exhibited a statistically significant higher gingival index and amount of lingual gingival recession compared to controls but they appear to be no more at risk in developing of periodontal disease [11, 14].

At the end, we recommend further studies using larger samples to investigate other periodontal statues in oral lichen planus with desquamative gingivitis.

## 5. Conclusion

Our results showed that periodontal status is worse in OLP patients with DG if compared with healthy controls.

## Figures and Tables

**Table 1:** Mean values of clinical periodontal parameters in each group.

Periodontal parameters	PI	GI	PD (mm)	BOP (%)	CAL (mm)
Control group	0.36 ± 0.18	0.05 ± 0.03	2.4 ± 0.2	2.8 ± 1.7	2.5 ± 0.28
Case group	0.78 ± 0.32	0.51 ± 0.38	3.02 ± 0.72	15.9 ± 8.42	3.5 ± 0.43

## References

[1] Canto AM, Muller H, Freitas RR, Santos PS (2010). Oral liehen (OLP): clinical and complementary diagnosis. *Anais Brasileiros de Dermatologia*.

[2] Axell T, Zain RB, Siwamogstham P, Tantiniran D, Thampipit J (1990). Prevalence of oral soft tissue lesions in out-patients at two Malaysian and Thai dental schools. *Community Dentistry and Oral Epidemiology*.

[3] Bánóczy J, Rigó O (1991). Prevalence study of oral precancerous lesions within a complex screening system in Hungary. *Community Dentistry and Oral Epidemiology*.

[4] Albrecht M, Banoczy J, Dinya E, Tamas G (1992). Occurrence of oral leukoplakia and lichen planus in diabetes mellitus. *Journal of Oral Pathology and Medicine*.

[5] Ismail SB, Kumar SK, Zain RB (2007). Oral lichen planus and lichenoid reactions: etiopathogenesis, diagnosis, management and malignant transformation. *Journal of Oral Science*.

[6] Scully C, Carrozzo M (2008). Oral mucosal disease: lichen planus. *British Journal of Oral and Maxillofacial Surgery*.

[7] Leao JC, Ingafou M, Khan A, Scully C, Porter S (2008). Desquamative gingivitis: retrospective analysis of disease associations of a large cohort. *Oral Diseases*.

[8] Lo Russo L, Guiglia R, Pizzo G (2010). Effect of desquamative gingivitis on periodontal status: a pilot study. *Oral Diseases*.

[9] Lo Russo L, Fedele S, Guiglia R (2008). Diagnostic pathways and clinical significance of desquamative gingivitis. *Journal of Periodontology*.

[10] Arduino PG, Farci V, D’Aiuto F (2011). Periodontal status in oral mucous membrane pemphigoid: initial results of a case-control study. *Oral Diseases*.

[11] Schellinck AE, Rees TD, Plemons JM, Kessler HP, Rivera-Hidalgo F, Solomon ES (2009). A comparison of the periodontal status in patients with mucous membrane pemphigoid: a 5-year follow-up. *Journal of Periodontology*.

[12] Akman A, Kacaroglu H, Yilmaz E, Alpsoy E (2008). Periodontal status in patients with pemphigus vulgaris. *Oral Diseases*.

[13] Newman MG, Takei HH, Carranza AF (2011). *Carranza's Clinical Periodontology*.

[14] Tricomo MB, Rees TD, Halmon WW, Wright JM, Cueva MA, Plemons JM (2006). Periodontal status in patients with gingival mucosal membrane pemphigoid. *Journal of Periodontology*.

